# KLRG1 restricts memory T cell antitumor immunity

**DOI:** 10.18632/oncotarget.11430

**Published:** 2016-08-20

**Authors:** Lei Li, Shanshan Wan, Kaixiong Tao, Guobin Wang, Ende Zhao

**Affiliations:** ^1^ Department of Gastrointestinal Surgery, Union Hospital, Tongji Medical College, Huazhong University of Science and Technology, Wuhan, Hubei, China; ^2^ Department of Surgery, University of Michigan, Ann Arbor, Michigan, USA

**Keywords:** KLRG1, senescence, memory T cells, antitumor immunity

## Abstract

Killer cell lectin-like receptor subfamily G member 1 (KLRG1) has been found on human memory T lymphocytes. However, the roles of KLRG1 on human T cells especially in tumor microenvironment have not been fully understood. Our results showed KLRG1 expression on T cells significantly increased in tumor microenvironment. KLRG1^+^ T cells exhibited poor proliferative capacity with decreased effector cytokine production. Meanwhile, KLRG1^+^ T cells expressed abundant pro-inflammatory cytokines and demonstrated high level of Foxp3 expression. KLRG1^+^ T cells showed decreased expression of miRNA-101 and higher expression of CtBP2. Our results indicated KLRG1 might contribute to the impaired antitumor immunity of memory T cells in tumor microenvironment. Thus, repressing KLRG1 on human memory T cells might be a novel therapeutics against cancer.

## INTRODUCTION

KLRG1 is a co-inhibitory receptor belonging to inhibitory killer cell lectin-like receptors on NK cells and antigen-experienced human T cells [1, 2]. KLRG1 was thought to be a senescent marker for human T cells with poor proliferation and impaired clonal expansion after stimulation [3]. It has been reported that the expression of KLRG1 on human CD8^+^ T cells was elevated in the condition of virus infection, which may contribute to increased morbidity of infectious diseases [1, 4]. However, the expression and role of KLRG1 on human T cells in the tumor microenvironment remain to be well addressed.

Programmed cell death protein 1 (PD-1, CD279) has been reported to be involved in the anergy and exhaustion of T cells [5, 6]. T-cell inhibitory receptor Tim-3 (T-cell immunoglobulin and mucin-domain containing-3) was reported to be an immune checkpoint receptor [7]. Tim-3 was also considered as a surface marker for T cell exhaustion [8, 9].

Ectonucleotidases play a pivotal role in the regulation of purinergic signaling pathway. CD39 (ectonucleoside triphosphate diphosphohydrolase 1, E-NTPDase1) can convert ATP into AMP, which is subsequently dephosphorylated into adenosine by CD73 (ecto-50-nucleotidase, Ecto50NTase) [10, 11]. The metabolic pathway shifts the ATP-driven proinflammatory environment to adenosine-induced anti-inflammatory status [10]. It tilts the balance towards immunosuppressive microenvironment [11], favoring tumor immune-evasion [12]. Extracellular accumulation of adenosine can lead to T cell inhibition and anergy [13, 14], contributing to tumor progression [15]. Antibodies targeting CD73 and CD39 exhibited proven efficacy in mouse tumor models [16]. CD73-adenosine pathway played a major role in the immune evasion and high CD73 expression predicted poor outcome of ovarian cancer patients [17].

MicroRNAs (miRNAs) are involved in the post-transcriptional regulation through binding to 3′ untranslated regions (UTRs) and coding region (CDS) of target mRNAs [18–20]. miRNAs are important functional modulators for T cells [21]. C-terminal binding protein-2 (CtBP2) is a transcriptional co-repressor gene involved in tumorigenesis and tumor progression [22, 23]. We have recently demonstrated that miRNA-101 increased cancer cell stemness by repressing CtBP2 [22].

In this study, we examined the roles of KLRG1 in human T cells and the underlying mechanism, especially in the tumor microenvironment.

## RESULTS

### KLRG1^+^ T cells enriched in tumor microenvironment

**Figure 1:**
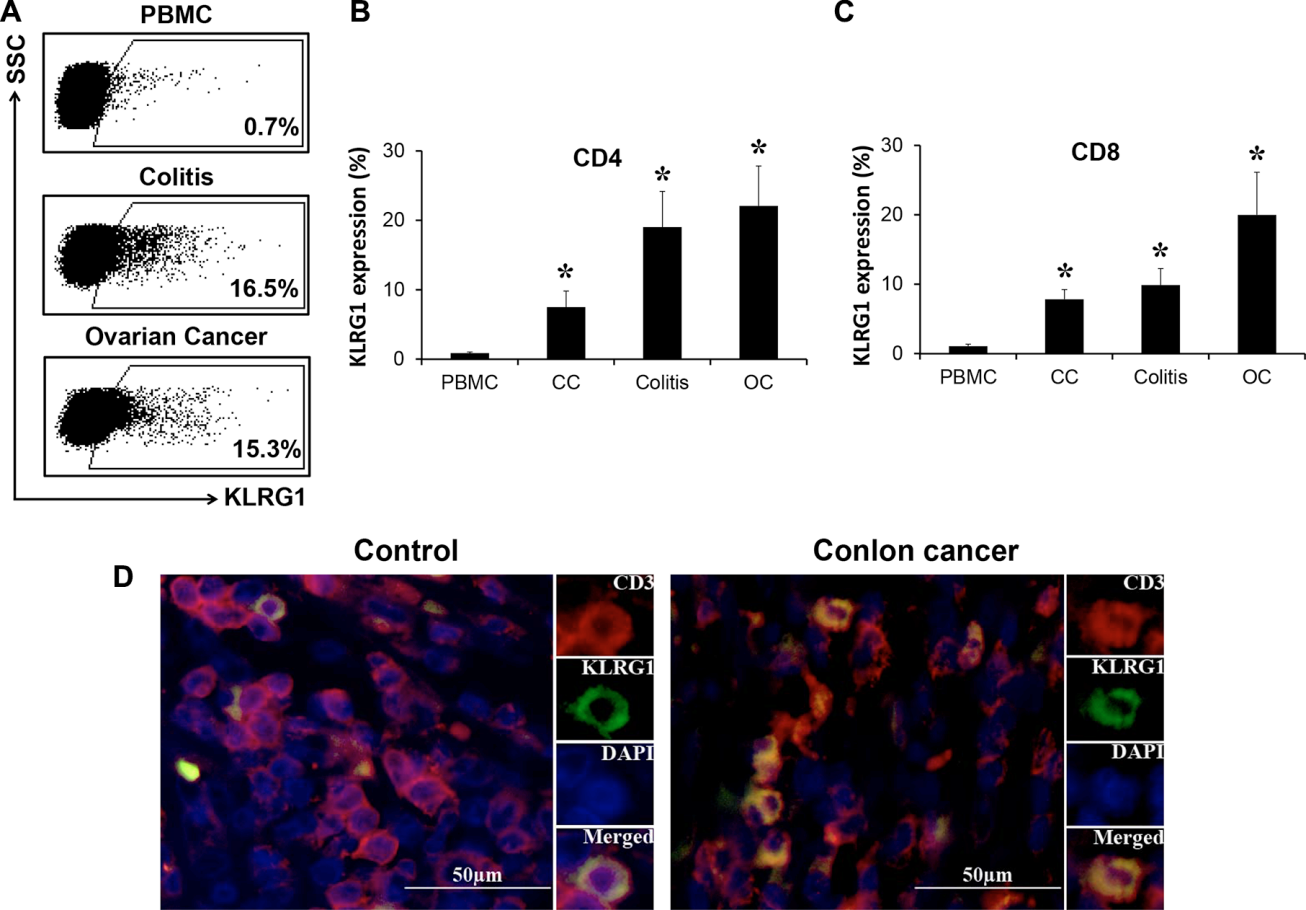
KLRG1^+^ T cells enriched in tumor microenvironment (**A**) KLRG1 expression on T cells from PBMC of healthy donors and T cells isolated from ovarian cancer and colitis tissue analyzed by flow cytometry. One of 8 representative data was shown. (**B**, **C**) KLRG1 expression on CD4 (B) and CD8 (C) T cells in colon cancer (CC), colitis and ovarian cancer (OC) by flow cytometry. *n* = 10, *p < 0.05. (**D**) KLRG1 (green) expression on CD3 (red) T cells in tissues from colon cancer patients and patients received colectomy after trauma by immunofluorescence staining. One of 4 representative data was shown.

We set out to check the expression level of KLRG1 on human T cells. We isolated peripheral blood mononuclear cells (PBMC) from healthy donors and checked KLRG1 expression by flow cytometry. It showed that CD3^+^ T cells expressed limited KLRG1 in healthy donors (Figure 1A). In contrast, T cells isolated from ovarian cancer and colitis expressed high level of KLRG1 (Figure 1A). We also found KLRG1 expression significantly increased in both CD4^+^ and CD8^+^ T cells in colon cancer, colitis and ovarian cancer (Figure 1B, 1C). Immunofluorescence cell staining confirmed the higher level of KLRG1 expression on CD3^+^ T cells in colon cancer tissue compared with the expression in colon tissue from patients received colectomy after trauma (Figure 1D). Similar results were observed in colitis and ovarian cancer tissues (data not shown). The results indicated that human T cells in tumor microenvironment expressed increased level of KLRG1.

### KLRG1^+^ T cells exhibited senescent characteristics

**Figure 2:**
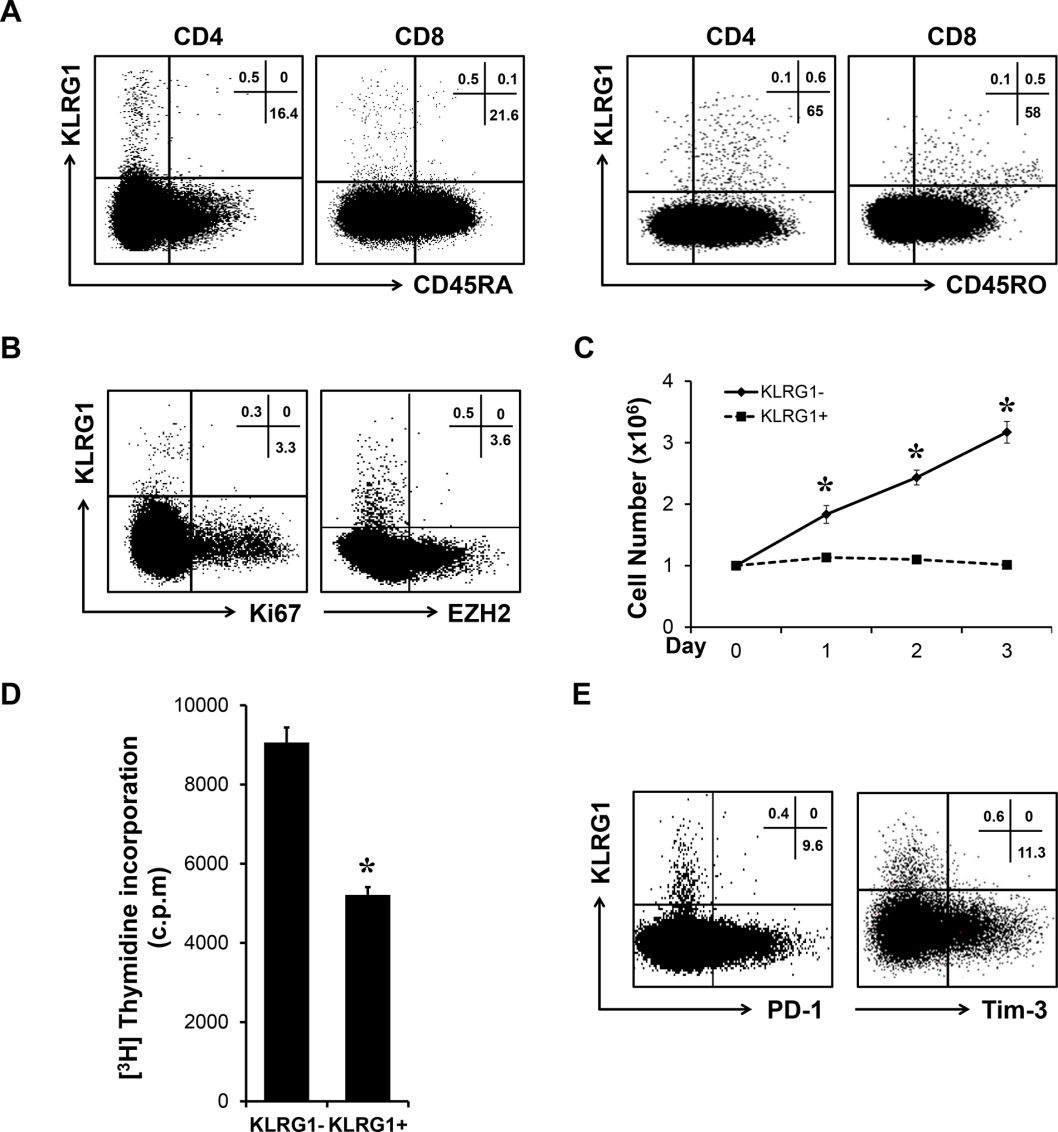
KLRG1^+^ T cells exhibited senescent characteristics (**A**) KLRG1 expression on T cells is majorly confined to CD45RA negative memory cells. One of 6 representative data was shown. (**B**) KLRG1^+^ T cells express low level of proliferative markers Ki67 and EZH2. One of 4 representative data was shown. (**C**) T cells were activated with antiCD3 and antiCD28 antibodies for 3 days. The cell number of each group was counted. *n* = 3, *p < 0.05. (**D**) FACS sorted KLRG1^+^ and KLRG1^−^ CD8 T cells were stimulated with antiCD3 and antiCD28 antibodies and 1:1 irradiated PBMC for 2 days and co-cultured with thymidine overnight. Thymidine incorporation of each group was examined. *n* = 3, *p < 0.05. (**E**) Relationship of KLRG1 with PD-1 and Tim-3 of CD8 T cells was accessed by flow cytometry. One of 5 representative data was shown.

We next investigated the characteristics of KLRG1^+^ T cells. Flow cytometry analysis presented KLRG1^+^ T cells were majorly localized in the CD45RA^−^ CD45RO^+^ population (Figure 2A), indicating KLRG1^+^ T cells possess memory phenotype. As memory T cells possess proliferative capacity due to previous antigen challenge. We examined whether KLRG1^+^ T cells could keep proliferative ability. Our results showed KLRG1^+^ T cells barely expressed proliferative marker Ki67 (Figure 2B). Our group have recently reported human memory T cells highly express epigenetic repressor EZH2 [24], which could be used as an alternative proliferative marker. Similarly, we found KLRG1^+^ T cells expressed limited EZH2 (Figure 2B), which indicated KLRG1^+^ T cells lost the proliferative capacity. To further confirm the limited proliferation of KLRG1^+^ T cells, we performed functional study via proliferation assay to compare FACS sorted KLRG1 positive and negative T cells. As shown in Figure 2C, KLRG1^−^ T cells proliferated efficiently after challenged with anti-CD3 and anti-CD28 antibodies while KLRG1^+^ cells showed poorly proliferative potential. Additionally, KLRG1^+^ T cells incorporated significantly less thymidine compared with KLRG1^−^ T cells (Figure 2D). It was recently reported that KLRG1 impairs T cell response in HCV infection via p16 and p27 pathways [25]. Interestingly, our results showed no significant differences of cyclin-dependent kinase inhibitors between KLRG1 positive and negative populations (Supplementary Figure S1A). Similary, we found no significant differences of cyclin-related genes (Supplementary Figure S1B).

To exclude the possibility that KLRG1^+^ T cells are functionally exhausted, we performed flow cytometry and found that KLRG1^+^ T cells were distinct from PD-1 or Tim-3 positive populations (Figure 2E). Further analysis showed no significant differences of Bcl-2 and Bcl-XL expression between KLRG1^+^ and KLRG1^−^ CD8^+^ T cells (Supplementary Figure S1C), indicating that KLRG1^+^ T cells were not undergoing apoptosis. These results indicated that human KLRG1^+^ T cells were terminally differentiated memory cells with senescent characteristics and limited proliferative potential.

### KLRG1 dampened T cell effector function

**Figure 3:**
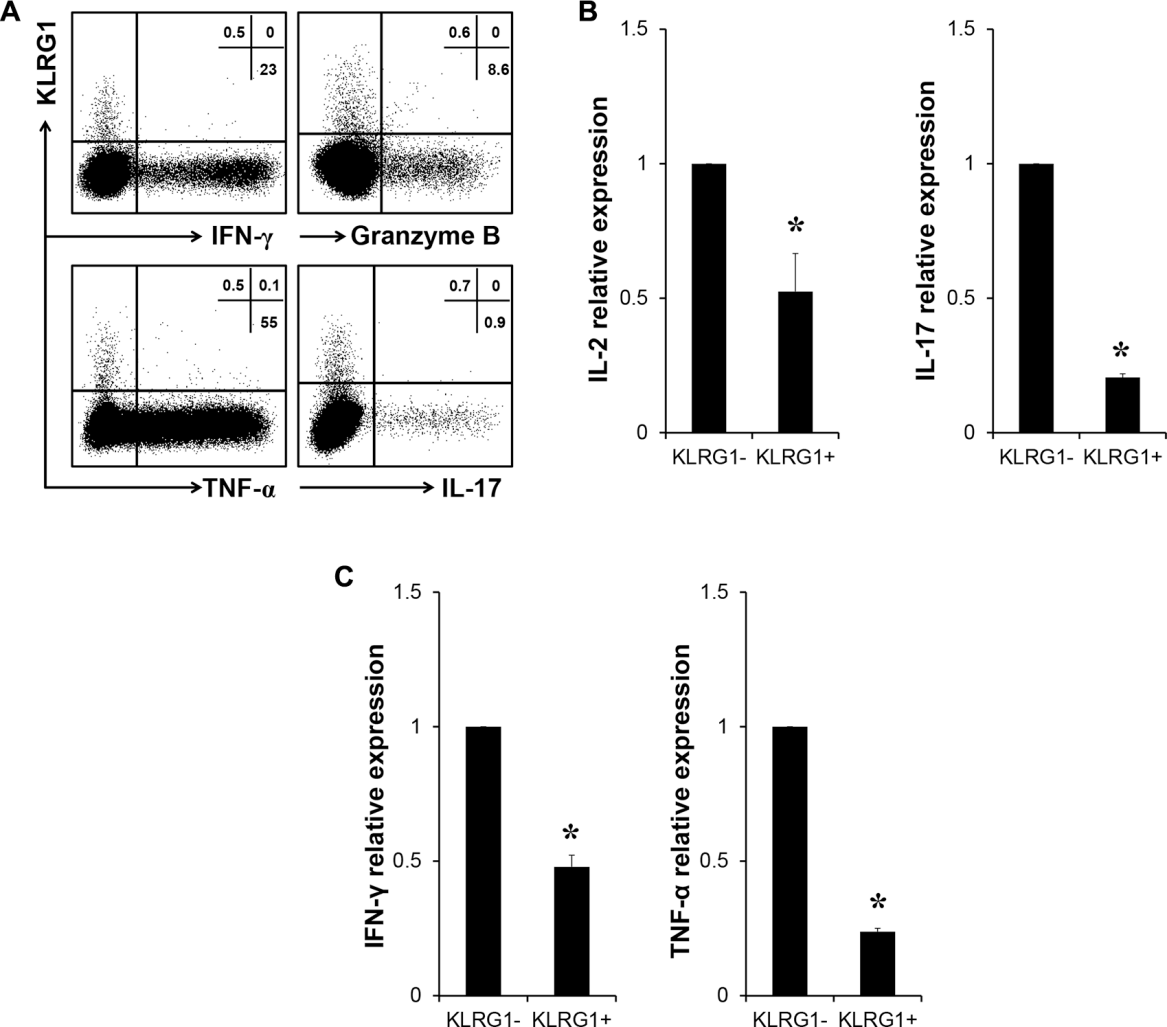
KLRG1 expression dampened T cell effector function (**A**) Relationship of KLRG1 expression with effector cytokines IFN-γ, Granzyme B and TNF-α of CD8 T cells and IL-17 of CD4 T cells. One of 4 representative data was shown. (**B**) Relative expression of IL-2 and IL-17 genes of FACS sorted KLRG1^+^ and KLRG1^−^ CD4 T cells by RT-PCR. *n* = 8, *p < 0.05. (**C**) Relative expression of IFN-γ and TNF-α genes of FACS sorted KLRG1^+^ and KLRG1^−^ CD8 T cells by RT-PCR. *n* = 5, *p < 0.05.

We have shown that human KLRG1^+^ T cells exhibited senescent phenotype. Next we examined the effector function of KLRG1^+^ T cells. Flow cytometry analysis showed that KLRG1^+^ CD4 T cells barely produced IL-17 and KLRG1^+^ CD8 T cells expressed limited effector cytokines IFN-γ, Granzyme B and TNF-α (Figure 3A). We FACS sorted KLRG1 positive and negative T cells from PBMC and performed RT-PCR analysis. Our results showed significantly less expression of IL-2 and IL-17 in KLRG1^+^ CD4 T cells (Figure 3B) and IFN-γ and TNF-α in KLRG1^+^ CD8 T cells compared with their KLRG1^−^ counterparts (Figure 3C). Thus, our results indicated that KLRG1 dampened T cell effector function.

### KLRG1^+^ T cells contributed to the immunosuppressive network in tumor microenvironment

**Figure 4:**
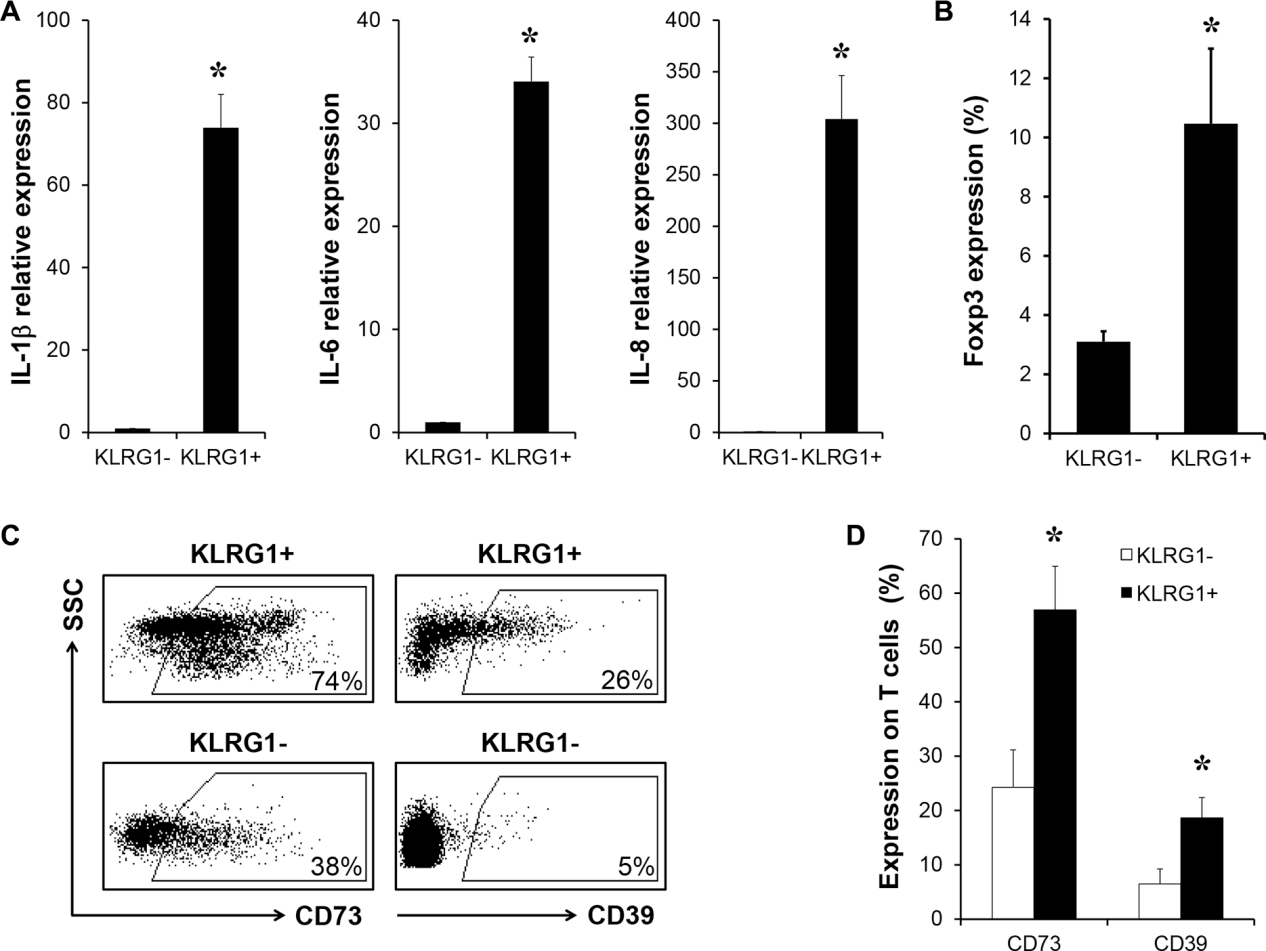
KLRG1^+^ T cells contributed to the immunosuppressive network in tumor microenvironment (**A**) Relative expression of pro-inflammatory cytokine genes IL-1b, IL-6 and IL-8 of FACS sorted KLRG1^+^ and KLRG1^−^ CD8 T cells by RT-PCR. *n* = 6, *p < 0.05. (**B**) Percentage of Foxp3 expressin in KLRG1^+^ CD4 T cell population was examined by flow cytometry. *n* = 4, *p < 0.05. (**C**) (**D**) Expression of ectonucleatidases CD73 and CD39 in human KLRG1^+^ memory CD8 T cells was analyzed by flow cytometry. One of 5 representative data was shown, *p < 0.05.

As shown above, KLRG1^+^ T cells have impaired effector cytokine production. Next we investigated the production of pro-inflammatory cytokines. We found KLRG1^+^ T cells secreted significantly higher levels of IL-1b, IL-6 and IL-8 than KLRG1^−^ T cells by RT-PCR analysis (Figure 4A). Furthermore, flow cytometry analysis discovered more Foxp3 positive cells in KLRG1^+^ CD4 T cell population (Figure 4B). Expression of ectonucleotidases CD73 and CD39 was reported to be immunosuppressive and the subsequent production of adenosine contributed to impaired anti-tumor T cell response [10, 11, 26]. Interestingly, our results uncovered that KLRG1^+^ memory CD8 T cells highly expressed CD73 and CD39 (Figure 4C and 4D). These results indicated that human KLRG1^+^ T cells possessed immune-suppressive function and pro-inflammatory potential, which may contribute to the immune-suppressive network in the tumor microenvironment.

### miRNA-101/CtBP2 pathway involved in KLRG1-restricted antitumor immunity

**Figure 5:**
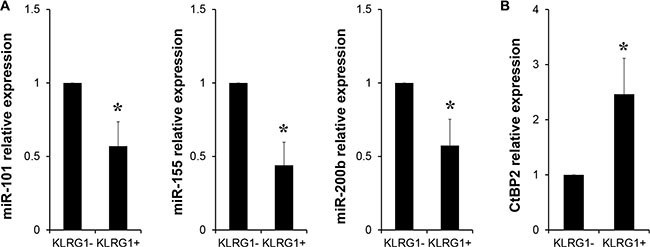
miRNA-101/CtBP2 pathway involved in KLRG1-restricted antitumor immunity (**A**) Relative expression of micro RNAs miR-101, miR-155 and miR-200b of FACS sorted KLRG1^+^ and KLRG1^−^ CD8 T cells by RT-PCR. *n* = 4, *p < 0.05. (**B**) Relative expression of corepressor gene CtBP2 of FACS sorted KLRG1^+^ and KLRG1^−^ CD8 T cells by RT-PCR. *n* = 5, *p < 0.05.

Then we investigated the underlying mechanism regulating KLRG1^+^ T cells. miRNAs are known to be critical for T cell regulation. Our results showed diminished expression of several miRNAs in KLRG1^+^ CD8 T cells compared with expression in KLRG1^−^ counterparts by RT-PCR analysis (Figure 5A). Among these reduced miRNAs, we focused on miRNA-101. We have reported that miRNA-101 increased cancer cell stemness by repressing the corepressor gene C-terminal binding protein-2 (CtBP2) [22]. In line with our previous findings, KLRG1^+^ CD8 T cells exhibited significantly elevated expression of CtBP2 (Figure 5B). Taken together, our results indicated miRNA-101 might contribute to impaired antitumor immunity restricted by KLRG1 through repressing CtBP2.

## DISCUSSION

KLRG1 is considered as a senescent marker for human T cells [3]. Consistent with the previous reports, our results showed that human KLRG1^+^ T cells exhibited poor proliferative potential. And KLRG1 expression was confined to memory population, indicating KLRG1^+^ T cells were antigen-experienced memory cells. Furthermore, KLRG1^+^ T cells were distinct from functionally exhausted or anergy populations. It was reported that mouse KLRG1^high^ CD8 T cells expressed lower levels of both anti-apoptotic Bcl-2 and pro-apoptotic Bim compared with KLRG1^low^ counterparts after LCMV infection [27]. However, our results exhibited no significant differences of Bcl-2 and Bcl-XL expression between human KLRG1^+^ and KLRG1^−^ CD8 T cells, suggesting KLRG1^+^ T cells presented no apoptosis preference. The results indicated KLRG1^+^ T cells were terminally differentiated memory T cells which haven't entered the apoptotic stage.

Human CD8 T cells from virus infection highly expressed KLRG1 which may be related with the increased incidence of infectious diseases [1, 4]. We found elevated KLRG1 expression on T cells from patients with cancer and autoimmune disease. It suggested that KLRG1 might contribute to the T cell dysfunction in pathological conditions. Our data suggested that except for the poor proliferative capacity, KLRG1 resulted in diminished production of effector cytokines and meanwhile elevated production of pro-inflammatory cytokines of T cells. It has been reported that KLRG1^+^ Treg cells elevated in a mouse model of experimental autoimmune encephalomyelitis (EAE) [28]. Our results presented elevated Foxp3 expression in KLRG1^+^ CD4 T cells, indicating KLRG1^+^ T cells may be endowed with immunosuppressive capacity. The roles of human KLRG1^+^ Treg cells in tumor microenvironment deserve to be further investigated.

Ectonucleotidases CD39 and CD73 were reported to impair immune system by switching from pro-inflammatory to the anti-inflammatory status [10]. The subsequent production of adenosine contributed to tumor progression by impairing T cell antitumor immunity [26]. Interestingly, we found significantly higher levels of both CD39 and CD73 expression on KLRG1^+^ CD8 T cells. Moreover, adenosinergic regulation was demonstrated to be associated with the induction of Treg cells and myeloid-derived suppressor cells (MDSCs) and inhibition of dendritic cells (DCs), contributing to T cell anergy and immunosuppression [14, 29, 30]. The results suggested human KLRG1^+^ T cells might be involved in the dysfunction of T cell antitumor immune response by regulating metabolic pathway through ectonucleotidases.

miRNAs have been reported to be an important modulator in T cell exhaustion in HIV infection [31]. However, little is known about the effect of miRNAs on human T cell senescence. In this work, we demonstrated that KLRG1^+^ CD8 T cells expressed lower levels of miR-101, miR-155 and miR-200b. miR-155 has been demonstrated to be required for effector CD8 T cell responses against infection and cancer [32]. We have previously reported that miRNA-101 increased cancer cell stemness by repressing CtBP2 [22]. Similarly, our data here exhibited elevated CtBP2 expression in KLRG1^+^ CD8 T cells. Moreover, CtBP2 has been reported to be a repressor of T cell cytokine production [33, 34], which could lead to the diminished production of effector cytokine of human KLRG1^+^ T cells. Thus, we hypothesized that declined miR-101 expression in human KLRG1^+^ T cells leaded to elevated expression of co-repressor CtBP2, which subsequently suppressed the effector cytokine production of KLRG1^+^ T cells, resulting in impaired T cell antitumor immunity in tumor microenvironment.

Taken together, our work suggested that KLRG1 was actively participating in the restricted T cell antitumor immunity by the following five aspects: 1) poor proliferative capacity, 2) diminished production of effector cytokines, 3) elevated production of pro-inflammatory cytokines, 4) immunosuppressive potential and 5) increased adenosine production by ectonucleotidase. And miR-101/CtBP2 pathway might be involved in the impaired antitumor immunity restricted by KLRG1. Therefore, our work suggested that repressing KLRG1 could be a novel therapeutics against tumor.

## MATERIALS AND METHODS

### Human subjects and human tissues

Peripheral blood was from healty donor and human primary CD3^+^ T cells were enriched from PBMC with RosetteSep enrichment cocktail following with Ficoll density gradient. Human T cells were cultured in RPMI 1640 with 10% FBS and 1% penicillin/streptomycin. Human tissues of colon cancer (*n* = 10), colitis (*n* = 10), ovarian cancer (*n* = 10) and traumatic colon (*n* = 4) were obtained from patients. A part of tissues were processed into frozen slides for immunofluorescent staining. Single suspension cells were obtained from chopped tissues by tissue digestion collagenase followed by density gradient centrifugation with Ficoll. Informed consents have been obtained from donors. The study was approved by the Institutional Review Boards of Union Hospital, Tongji Medical College, Huazhong University of Science and Technology.

### Fluorescence activated cell sorting (FACS) and flow cytometry analysis

For cytokine detection, cells were stimulated with phorbol myristate acetate (50 ng/mL; Sigma-Aldrich), ionomycin (1 μM; Sigma-Aldrich) for 4 hours with protein transport inhibitor (GolgiPlug 1 μl/ml, GolgiStop 2/3 μl/ml; BD Bioscience) in complete medium (CM) before staining. Cells were first stained extracellularly with specific antibodies against human CD3, CD4, CD8, CD45RA, CD45RO (BD Bioscience), then were fixed and permeabilized with Fixation/Permeabilization solution (eBioscience), and finally were stained intracellularly with anti-IL-2, anti-IL-17, anti-tumor necrosis factor-α (TNF-α), anti-interferon-γ (IFN-γ), anti-Granzyme B, anti-Ki67 and anti-EZH2 (BD Biosciences). Samples were acquired on flow cytometry (LSR II; Becton Dickinson) and data were analyzed with DIVA software (BD Biosciences) [35]. The gating strategy was shown in Supplementary Figure S2. KLRG1^+^ and KLRG1^−^ T cells were purified and sorted with FACSAria cell sorter (Becton Dickinson).

### [^3^H] Thymidine incorporation assay

The Thymidine incorporation assay was done as described [6, 36]. Briefly, CD3 T cells were seeded into 96-well plate with serial dilution and stimulated with 1:1 irradiated human PBMC, 2.5 μg/ml rhαCD3 and 1.25 μg/ml rhαCD28 for 2 days in 37°C incubator in 100 μl CM. Add 1 μCi/well [^3^H] thymidine diluted in 100 ul CM and culture overnight in 37°C incubator. Then put the plate in −80°C freezer for 1 day. Thaw at room temperature and harvest the cells onto a glass fibre filter. Dry up the filter and immerse into Betaplate Scint (PerkinElmer) then seal it into a plastic bag after removing the extra scint fluid. Then count the liquid scintillation with a MicroBeta Jet machine (PerkinElmer). There was no stimulation for Jurkat cells. In some cases, 5 μM DZNep was added into the cell culture when indicated.

### RT-PCR

RNA was isolated with RNeasy Mini Spin Column (Qiagen) or Trizol (Invitrogen). And reverse transcription was performed with AMV cDNA synthesis kit (Invitrogen). Briefly, 10 μl RNA and 1 μl Oligo(dT)_20_ were incubated for 5 minutes at 65°C on Thermomixer (eppendorf). Then 4 μl 5× cDNA synthesis buffer, 2 μl dNTP, 1 μl DTT, 1 μl RNase OUT and 1 μl AMV RTase were added and co-incubated for 1 hour at 50°C. Finally the mixture was incubated for 5 minutes at 85°C. Quantitative RT-PCR was performed with Fast SYBR Green Master Mix (Applied Biosystems) on Mastercycler (eppendorf). TaqMan microRNA assay kits (Applied Biosystems) were used for microRNA detection. The relative expression was acquired by normalizing to the expression of human b-actin with the formula of 2^−ΔCt^. For miRNA detection, relative expression was normalized to U6. Detail of primers is summarized in Supplementary Table S1.

### Immunofluorescent staining

Frozen sections from colitis, ovarian cancer, colon cancer and traumatic colon were fixed with 2% paraformaldehyde and permeabilized for 1 hour at RT with PBS with 0.1% Triton X-100. Slides were incubated with primary antibodies against KLRG1 (Santa Cruz Biotechnology, sc-23598, 1:100) or CD3 (Dako, A0452, 1:100) for overnight at 4°C after blocking with 10% goat serum. After washing away primary antibodies, slides were incubated with fluorescence-conjugated secondary antibodies for 1 hour at RT. Then staining was examined under fluorescent microscope (Leica DM5000 B).

### Statistics

Dependent on data distribution and experimental design, paired or unpaired Student *t* test and Mann-Whitney *U* tests were used. All analyses were done by using SAS 9.3 software. The results were considered statistically significant when *p* value is less than 0.05.

## SUPPLEMENTARY MATERIALS FIGURE AND TABLE


