# Supplementary material for: KLRG1 restricts memory T cell antitumor immunity
Source: Oncotarget. 2016 Aug 20;7(38):61670–8. doi: 10.18632/oncotarget.11430 (PMC5308681; doi:10.18632/oncotarget.11430)
Supplement: Supplementary file 1 [file oncotarget-07-61670-s001.pdf]

KLRG1 restricts memory T cell antitumor immunity

Supplementary Materials

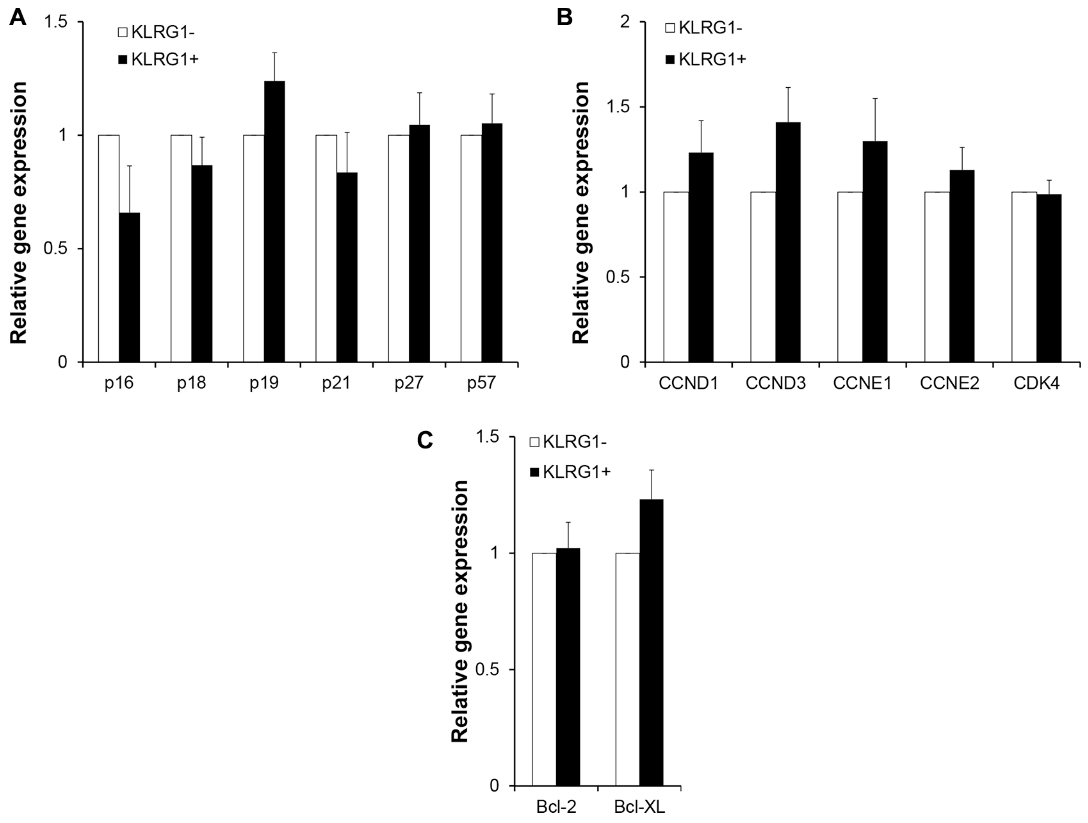

Supplementary Figure S1: KLRG1$^+$ T cells exhibited senescent characteristics. (**A**) Relative expression of cyclin-dependent kinase inhibitors genes p16, p18, p19, p21, p27 and p57 of FACS sorted KLRG1$^+$ and KLRG1$^-$ CD8 T cells was examined by RT-PCR. *n* = 4. (**B**) Relative expression of cyclin-related genes CCND1, CCND3, CCNE1, CCNE2 and CDK4 of FACS sorted KLRG1$^+$ and KLRG1$^-$ CD8 T cells was examined by RT-PCR. *n* = 4. (**C**) Relative expression of Bcl-2 and Bcl-XL genes of FACS sorted KLRG1$^+$ and KLRG1$^-$ CD8 T cells were examined by RT-PCR. *n* = 4.

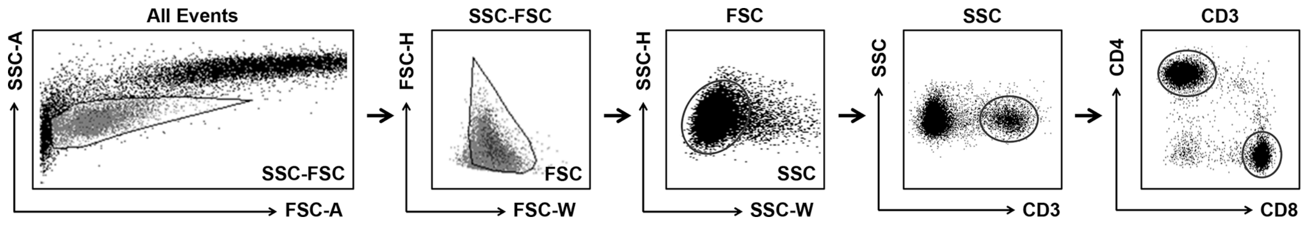

Supplementary Figure S2: Gating strategy for the flow cytometric analysis.

Supplementary Table S1: Primer information

Name	Sense	Anti-sense
p16	ATGCCGCGGAAGGTCCCTCA	AAAGCGGGGTGGGTTGTGGC
p18	GGAACGAGTTGGCGTCCGCA	GGAAACCTGCTCTGGCCGCA
p19	GAGCTGGTGCATCCCGACG	GGGCAGGAGAAACAAGAAGAGAAAG
p21	GCCCAGTGGACAGCGAGCAG	GCCGGCGTTTGGAGTGGTAGA
p27	GCGACCTGCAACCGACGATTCT	GAGGCCAGGCTTCTTGGGCG
p57	CTGACCAGCTGCACTCGGGGATTTC	GCCGCCGGTTGCTGCTACATGA
CCND1	GGCCATGCTGAAGGCGGAGG	GCTCCAGCGACAGGAAGCGG
CCND3	CCTCCAAGCTGCGCGAGACC	GGCAGAGAGAGCCGGTGCAG
CCNE1	AGCCCCATCATGCCGAGGGA	TGGGGATCAGGGAGCAGGGG
CCNE2	TGAGCCGAGCGGTAGCTGGT	GGGATTCCGTCTGGCTGGGC
CDK4	CACTCTGGTACCGAGCTCCCGA	GGCTCCACGGGGCAGGGATA
Bcl-2	TGTGGAGAGCGTCAACCGGGAG	GCAAGCTCCCACCAGGGCCAAA
Bcl-XL	GCAAGCTCCCACCAGGGCCAAA	TGTGGAGAGCGTCAACCGGGAG
IL-2	GCACTTGTCACAAACAGTGCACCTAC	CAGTTCTGTGGCCTTCTTGGGCA
IL-17	ATGACTCCTGGGAAGACCTCATTG	TTAGGCCACATGGTGGACAATCGG
IFN-γ	AGCATCCAAAAGAGTGTGGAGACCA	AGCTGCTGGCGACAGTTCAGC
TNF-α	GTGATCGGCCCCCAGAGGGA	CACGCCATTGGCCAGGAGGG
IL-1b	AGCACCTCTCAAGCAGAAAACAT	TTGCATGGTGAAGTCAGTTATATCC
IL-6	AGGAACTCCTTAAAGCTGCG	CTTCGGTCCAGTTGCCTTCT
IL-8	ACATGACTTCCAAGCTGGCCGT	TTCCTTGGGGTCCAGACAGAGCT
CtBP2	GTCCGGGACGAGGGTTTCATCACC	AGGGGAACTTGCAGGAGTCTGC
β-actin	AGAGCCTCGCCTTTGCCGATCC	CTGGGCCTCGTCGCCCACATA
